# Vortex Beam Encoded All-Optical Logic Gates Based on Nano-Ring Plasmonic Antennas

**DOI:** 10.3390/nano9121649

**Published:** 2019-11-20

**Authors:** Houquan Liu, Hongchang Deng, Shijie Deng, Chuanxin Teng, Ming Chen, Libo Yuan

**Affiliations:** 1 Photonics Research Center, School of Electronic Engineering and Automation, Guilin University of Electronics Technology, Guilin 541004, China; sdeng_guet@163.com (S.D.); xinchuanteng@126.com (C.T.); m_chen@126.com (M.C.); lbyuan@vip.sina.com (L.Y.); 2 Guangxi Key Laboratory of Optoelectronic Information Processing, Guilin University of Electronics Technology, Guilin 541004, China

**Keywords:** all-optical logic gates, vortex beam, plasmonic

## Abstract

Vortex beam encoded all-optical logic gates are suggested to be very important in future information processing. However, within current logic devices, only a few are encoded by using vortex beams and, in these devices, some space optical elements with big footprints (mirror, dove prism and pentaprism) are indispensable components, which is not conducive to device integration. In this paper, an integrated vortex beam encoded all-optical logic gate based on a nano-ring plasmonic antenna is proposed. In our scheme, by defining the two circular polarization states of the input vortex beams as the input logic states and the normalized intensity of the plasmonic field at the center of the nano-ring as the output logic states, OR and AND (NOR and NAND) logic gates are realized when two 1st (1st) order vortex beams are chosen as the two input signals; and a NOT logic gate is obtained when one 1st order vortex beam is chosen as the input signal. In addition, by defining the two linear polarization states (*x* and *y* polarization) of the input vortex beams as the two input logic states, an XNOR logic gate is realized when two 1st order vortex beams are chosen as the two input signals.

## 1. Introduction

In the era of big data, the exchange of network data can only match the large capacity of data transmission if ultra-high speed is achieved. All-optical computing is an effective way to improve the data calculation and the rate of data exchange [1]. All-optical logic gates are key elements in all-optical computing and optical circuits [2]. Therefore, many schemes for optical logic gates have been proposed. These optical logic gates can be divided into two categories: one is based on nonlinear optics [3,4,5,6], and the other is based on linear coherence [7,8,9]. In comparison, the nonlinear optics-based optical logic gates depend on the small nonlinear susceptibility of conventional materials so that intense light power is needed, which is an obstacle for practical applications. Linear interference logic operation is achieved by the destructive or constructive interference of the two input signals in which the relative phase difference of the two input signals plays the key role, hence it can be realized under very low light power, even under single photon level [10]. In recent years, linear interference logic devices have aroused great interest among researchers.

In all-optical logic gates, the two input signals are two orthogonal light modes. In linear interference schemes, two path modes that propagate along different paths are usually used as the two input signal lights. Examples include the two counter-propagating incident beams in the coherent absorption schemes of a standing wave on ultrathin metamaterials [11,12]; the two surface plasmonic waves confined and guided in different plasmonic waveguides [13,14,15,16,17,18]; and the two light signals propagated in the two arms of an interferometer [19] or two different silicon waveguides of the optical ring/racetrack devices [20]. In addition to path modes, the two input signal lights can also be orthogonal light modes on other degrees of freedom, such as polarization and spatial modes (vortex beams, vector beams, fiber modes and so on). Using these degrees of freedom to construct all-optical logic gates, some beneficial performance can be obtained beyond the path coherence of logic gates. For example, encoding logic states on slot plasmonic nanoantenna by using polarization states can significantly reduce the size of the logic devices to a 300-nm scale [21]. To the best of our knowledge, only a few works use polarization modes [21] and vortex beams [22,23] as the control lights of all-optical logic gates.

A vortex beam is also named the orbital angular momentum (OAM) beam. This kind of beam has been widely considered [24,25,26,27] since Allen’s pioneering work in 1992 [28] due to its unique properties. It can be generated by using spatial light modulators [29], a q-plate [30], a spiral phase plate [31], metasurface [32], etc. One significant property of a vortex beam is that a high dimensional Hilbert space can be constructed by using its OAM state to achieve high-dimensional information encoding [33], which suggests it has great potential in future information processing (FIP). Therefore, in recent years, researchers have made great efforts to develop OAM-related technology for FIP, such as OAM multiplexing [34] and demultiplexing [35,36] technologies for large capacity data transmission, and integrated compact vortex beam emitters [37,38,39]. These imply that vortex beams may be key ingredients in FIP. However, unfortunately, most current logic devices are not compatible with vortex beams. This means that mode transformation of a vortex mode should be performed before it can be used for logical operations in future vortex beam-based all-optical computing. This will inevitably increase the complexity and cost of the system. Therefore, developing vortex beams encoded all-optical logic devices is suggested to be important, although there are already some vortex beams encoded logic devices [22,23]. Some space optical elements with big footprints (such as mirror, dove prism and pentaprism) are indispensable components, which is not conducive to device integration. Plasmonic devices are an effective solution for device miniaturization and integration. To the best of our knowledge, there are no reported vortex beams encoded plasmonic-based all-optical logic gates. In this paper, we put forward a vortex beams encoded scheme to achieve all-optical logic gates based on nano-ring plasmonic antennas.

A nano-ring plasmonic antenna is a very simple nano-structure. It can match the circular intensity distribution of vortex beams as long as they are aligned with each other, hence it can receive complete topology information (the phase variation along the azimuth angular) of the vortex beams. In this regard, in designing vortex beam-related devices, the nano-ring plasmonic antenna is superior to other kind of plasmonic antennas, such as the plasmonic waveguide antennas of previous all-optical logic devices [13,14,15,16,17,18]. The receive ports of these previous antennas are either straight nano slots, straight nano slot arrays, nano points or triangular nanostructures, and none match the circular intensity distribution of vortex beams (the reason why previous plasmonic-based logic devices are not compatible with vortex beams). In our scheme, OR, AND, NOT, NOR, NAND and XNOR logic gates are demonstrated theoretically via numerical calculations. In the following, we show them in detail.

## 2. Interactions between Light and Surface Plasmon Polaritons (SPPs) on Nano-Ring Plasmonic Antennas

**Figure 1:**
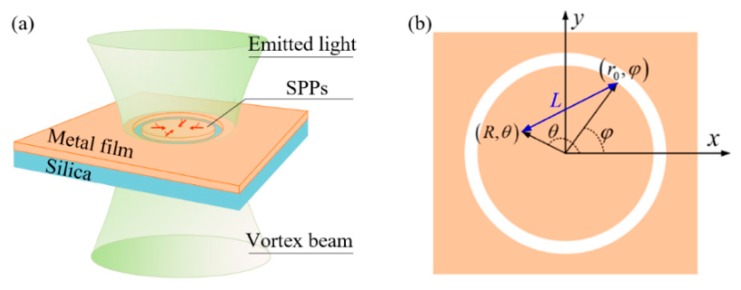
The diagram of a single ring plasmonic antenna. (**a**) is a 3D drawing of the nano-ring plasmonic antenna. (**b**) is a top view of the nano-ring plasmonic antenna and the coordinates for our calculation.

To show our scheme in detail, we first give the theory of the interactions between light and surface plasmon polaritons (SPPs) on nano-ring plasmonic antennas. A nano-ring plasmonic antenna is a structure that allows us to derive an analytical expression for the plasmonic focal field, which is well studied in [40]. Our following description of the theory follows the derivation process of [40]. The 3D drawing and the top view of the nano-ring plasmonic antenna and the coordinates for our calculation are illustrated in Figure 1. A single ring slot is etched into a thin metal film deposited on silica substrate. To fabricate this device, one should first plate the metal film on the silica substrate, then fabricate the ring slot by using focused ion beam lithography. Light illuminates the structure normally from the silica substrate side. Considering the incident light possessing both spin angular momentum (SAM) with spin quantum number σ and OAM with topological charge *l*, it can be expressed in cylindrical coordinates as

(1) $$E_{in}=e^{i\left(l+\sigma \right)\varphi }\left({\vec{e}}_{r}+i\sigma {\vec{e}}_{\varphi }\right),$$

where $i={\left(-1\right)}^{1/2}$ is the imaginary unit, ${\vec{e}}_{r}$ and ${\vec{e}}_{\varphi }$ are the radial unit vector and angular unit vector respectively. According to the excitation conditions of SPPs, when the slot is sufficiently narrow, only the radial component of the incident light can couple to the SPPs. Thus, the SPPs are exited along an incremental length of the annular slot at point $\left(r_{0},\varphi \right)$ which contributes to the plasmonic field at an observation point (R,θ) as a secondary source to generate a field increment given by

(2) $$d{\vec{E}}_{SPP}={\vec{e}}_{z}E_{0}e^{-k_{z}z}e^{i\left(l+\sigma \right)\varphi }e^{ik_{r}L}r_{0}d\varphi ,$$

where $E_{0}$ is a constant that is related to the coupling efficiency from the incident light to the SPPs, $k_{r}$ is the wave vector of the SPPs that propagate in the metal plane, and $k_{z}$ is the wave vector of the SPPs that propagate in the *z*-direction. In this paper, for simplicity, we ignore the propagation loss of the SPPs propagating in the metal plane, hence $k_{r}=2\pi /\lambda_{SPP}$ with $\lambda_{SPP}$ being the wave length of the plasma wave. If the observation point is near the origin point, we have $R\ll r_{0}$ (for example $R\leq 2\lambda_{SPP}$ when $r_{0}=10\lambda_{SPP}$). In this case it can be obtained that

(3) $$L={\left[r_{0}^{2}+R^{2}-2r_{0}R\cos(\theta -\varphi )\right]}^{1/2}\approx r_{0}-R\cos(\theta -\varphi ).$$

Hence, the total plasmonic field at the observation point is given by

(4) $$\begin{array}{l} {\vec{E}}_{SPP}\left(R,\theta \right)={\vec{e}}_{z}E_{0}e^{-k_{z}z}e^{ik_{r}r_{0}}r_{0}\int_{0}^{2\pi }e^{i\left(l+\sigma \right)\varphi }e^{-ik_{r}R\cos\left(\theta -\varphi \right)}d\varphi \\ \;\;={\vec{e}}_{z}2\pi i^{\left(l+\sigma \right)}E_{0}r_{0}e^{-k_{z}z}e^{ik_{r}r_{0}}J_{l+\sigma }\left(-k_{r}R\right)e^{i\left(l+\sigma \right)\theta }. \end{array}$$

Using Equation (4), we can calculate the intensity of the plasmonic field near the center of the nano-ring. It is determined by the total angular momentum. Here we should note that the above theoretical results are general. They will not be restricted by the particular material and thickness of the metal film, slot width, and light wavelength. Although these parameters could affect the coupling efficiency from the incident vortex beam to the SPPs and the propagation loss of the SPPs propagating in the metal plane, their effects can be condensed into the amplitude $E_{0}$. Hence, they do not affect the intensity distributions normalized by $|E_{0}{|}^{2}$.

## 3. Design and Discussion

In this section, according to the above theory derivation, we will show how to construct all-optical logic gates by using vortex beams as the input signals and the different intensity of the SPPs at the center point of the nano-ring as the output logic states.

### 3.1. OR and AND Logic Gates

**Figure 2:**
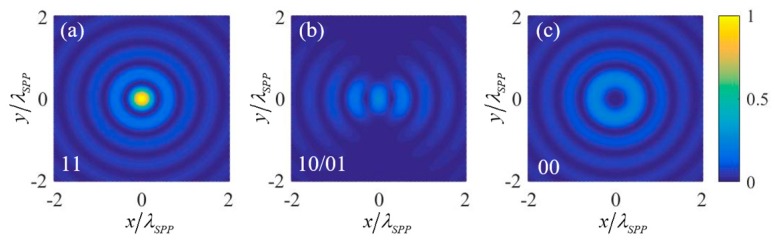
The normalized intensity of the plasmonic field near the center of the nano-ring for (**a**) input logic states “11”, (**b**) input logic states “10/01”, and (**c**) input logic states “00” in the realization scheme of OR and AND logic gates.

To realize OR and AND logic gates, two 1st order OAM beams (with *l* = −1) with the same amplitudes and initial phases are chosen as the two input signals. Similar to [21], the circular polarization states of the input OAM beams are utilized to denote the two input logic states. Right circular polarization (RCP) with σ=−1 and left circular polarization (LCP) with σ=1 are defined as the input states “0” and “1” respectively. Under these definitions, the intensity of the plasmonic field near the center of the nano-ring for four input states “11” (two RCP incidences), “10/01” (one RCP and one LCP incidences) and “00” (two LCP incidences) can be calculated. The results are shown in Figure 2, where (a), (b) and (c) correspond to the input logic states “11”, “10/01”, and “00” respectively. It is noted that the results are normalized by the intensity of the center point of the nano-ring under the “11” input state. Extracting the normalized intensity of the center point under the four input logic states and putting them into Table 1, it can be seen that the OR logic gate is obtained by setting the relative intensity threshold within the range of 0–0.25 and using the intensity lower and higher than the threshold to denote the two output logic states “0” and “1” respectively, and the AND logic gate is obtained by setting the relative intensity threshold within the range of 0.25–1.

The physical mechanism can be explained as follows: under the excitation, the plasmonic field near the center of the nano-ring is (l+σ)th order vortex. Under *l* = −1, the plasmonic field resulting from the “0” input state of an input signal is $-{\vec{e}}_{z}2\pi E_{0}r_{0}e^{-k_{z}z}e^{ik_{r}r_{0}}J_{-2}\left(-k_{r}R\right)e^{-i2\theta }$. It is a 2nd order vortex whose intensity at the center point is zero. The plasmonic field resulting from “1” input state of an input signal is then ${\vec{e}}_{z}2\pi E_{0}r_{0}e^{-k_{z}z}e^{ik_{r}r_{0}}J_{0}\left(-k_{r}R\right)$. It contributes to the plasmonic field of the center point by $\vec{F}={\vec{e}}_{z}2\pi E_{0}r_{0}e^{-k_{z}z}e^{ik_{r}r_{0}}J_{0}\left(0\right)$. Since the excitation of SPPs is a linear process, the center point plasmonic field should be $2\vec{F}$ when both the two input states are “1”. Therefore, the intensity of the plasmonic field at the center point under input states “11”, “10/01”, and “00” are respective $4|F{|}^{2}$, $|F{|}^{2}$ and 0, corresponding to normalized intensity 1, 0.25 and 0, respectively.

### 3.2. NOT Logic Gate

**Figure 3:**
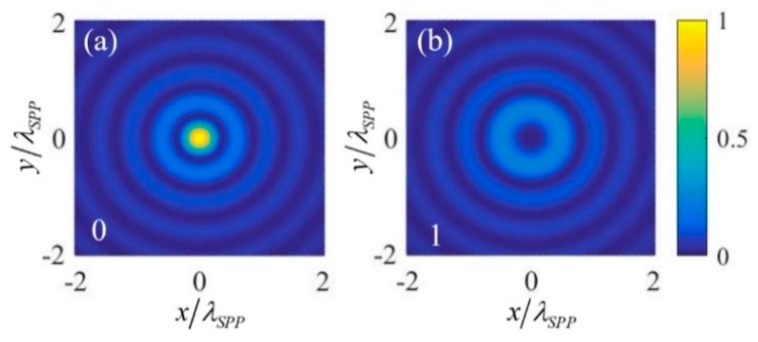
The normalized intensity of the plasmonic field near the center of the nano-ring for (**a**) input state “0” and (**b**) input state “1” in the realization scheme of NOT logic gate.

The NOT gate has only one input. To realize the NOT gate, the 1st order OAM beam (with *l* = 1) is chosen as the input signal. Similarly, the circular polarization states RCP (with σ=−1) and LCP (with σ=1) are defined as the input logic states “0” and “1” respectively. The calculated intensity of the plasmonic field near the center of the nano-ring for the two input states is shown in Figure 3, which is normalized by the intensity of the center point of the nano-ring under the “0” input state. Figure 3a,b are the results corresponding to the input logic states “0” and “1”, respectively. The normalized intensity of the center point under the two input logic states are extracted out and put into Table 2. It can be seen that if denoting the two output logic states “0” and “1” with normalized intensity 0 and 1, NOT logic gate is obtained. The physical mechanism is that under *l* = 1, the plasmonic field resulting from “1” input state is a 2nd order vortex (l+σ=1+1=2) whose intensity at the center point is zero, while plasmonic field resulting from “0” input state is ${\vec{e}}_{z}2\pi E_{0}r_{0}e^{-k_{z}z}e^{ik_{r}r_{0}}J_{0}\left(-k_{r}R\right)$, which contributes to the plasmonic field of the center point by $\vec{F}={\vec{e}}_{z}2\pi E_{0}r_{0}e^{-k_{z}z}e^{ik_{r}r_{0}}J_{0}\left(0\right)$. Thus, through normalized by $|F{|}^{2}$, the normalized intensity of the center point corresponding to the input logic states “0” and “1” are 1 and 0, respectively.

### 3.3. NOR and NAND Logic Gates

**Figure 4:**
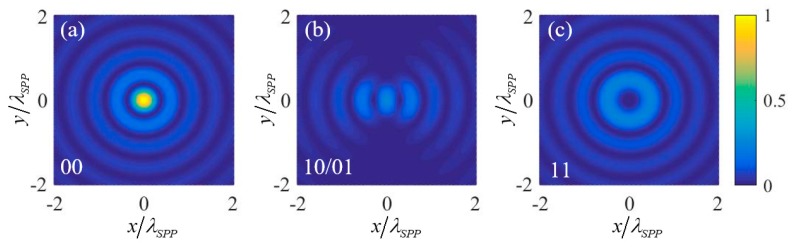
The normalized intensity of the plasmonic field near the center of the nano-ring for (**a**) input logic states “00”, (**b**) input logic states “10/01”, and (**c**) input logic states “11” in the realization scheme of NOR and NAND logic gates.

To realize NOR and NAND logic gates, two 1st order OAM beams (with *l* = 1) with the same amplitudes and initial phases are chosen as the two input signals. The circular polarization states RCP (with σ=−1) and LCP (with σ=1) are defined as the input logic states “0” and “1” respectively. The calculated intensity of the plasmonic field near the center of the nano-ring for four input logic states “11” “10/01”, and “00” is given in Figure 4, which is normalized by the intensity of the center point of the nano-ring under the “00” input state. Figure 4a–c correspond to the input logic states “00”, “10/01”, and “11” respectively. The normalized intensity of the center point under the four input logic states is extracted out and put into Table 3. It can be seen that the NOR logic gate is obtained by setting the relative intensity threshold within the range of 0.25–1 and using the intensity lower and higher than the threshold to denote the two output logic states “0” and “1” respectively, and the NAND logic gate is obtained by setting the relative intensity threshold within the range of 0–0.25.

Similarly, the physical mechanism here is that under *l* = 1, the plasmonic field resulting from the “1” input state is a 2nd order vortex (l+σ=1+1=2) whose intensity at the center point is zero, and the plasmonic field resulting from the “0” input state of an input signal is ${\vec{e}}_{z}2\pi E_{0}r_{0}e^{-k_{z}z}e^{ik_{r}r_{0}}J_{0}\left(-k_{r}R\right)$, which contributes to the plasmonic field of the center point by $\vec{F}={\vec{e}}_{z}2\pi E_{0}r_{0}e^{-k_{z}z}e^{ik_{r}r_{0}}J_{0}\left(0\right)$. So, the plasmonic field at the center point under input states “00”, “10/01”, and “11” are respective $2\vec{F}$, $\vec{F}$, and 0, corresponding to normalized intensity 1, 0.25, and 0, respectively.

### 3.4. XNOR Logic Gate

**Figure 5:**
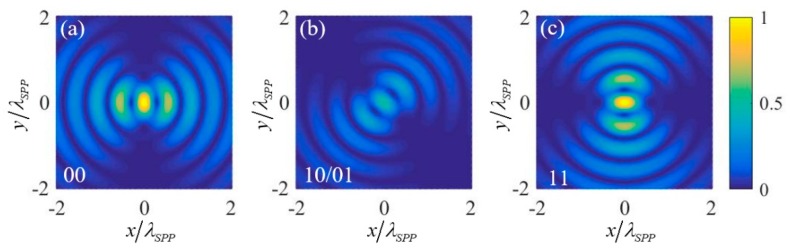
The normalized intensity of the plasmonic field near the center of the nano-ring for (**a**) input logic states “00”, (**b**) input logic states “10/01”, and (**c**) input logic states “11” in the realization scheme of XNOR logic gate.

To realize the XNOR logic gate, two 1st order OAM beams (with *l* = 1) with the same amplitudes and initial phases are chosen as the two input signals. Two linear polarization states are used to represent the two input logic states. The *x* axis linear polarization denotes input logic state “0” and the *y* axis linear polarization denotes input logic state “1”. These two linear polarization states can be expanded as superpositions of the two circularly polarization states, i.e., ${\hat{e}}_{x}=({\hat{e}}_{+}+{\hat{e}}_{-})/\sqrt{2}$ and ${\hat{e}}_{y}=-i({\hat{e}}_{+}-{\hat{e}}_{-})/\sqrt{2}$, where ${\hat{e}}_{x},{\hat{e}}_{y},{\hat{e}}_{+}$ and ${\hat{e}}_{-}$ are the unit vectors of *x* axis linear polarization, *y* axis linear polarization, LCP and RCP respectively. Thus, we can still calculate the intensity of the plasmonic field near the center of the nano-ring for the four input logic states “11” “10/01”, and “00” according to Equation (4). The results are shown in Figure 5, which are normalized by the intensity of the center point of the nano-ring under the “00” input state. Figure 5a–c correspond to the input logic states “00”, “10/01”, and “11”, respectively. The normalized intensity of the center point under the four input logic states are extracted out and put into Table 4. It can be seen that the XNOR logic gate is obtained if setting the relative intensity threshold within the range of 0.5–1 and using the intensity lower and higher than the threshold to denote the two output logic states “0” and “1” respectively.

The physical mechanism is explained as follows: firstly, the plasmonic field resulting from the “0” input state of an input signal is ${\vec{e}}_{z}\sqrt{2}\pi E_{0}r_{0}e^{-k_{z}z}e^{ik_{r}r_{0}}[J_{0}\left(-k_{r}R\right)-J_{2}\left(-k_{r}R\right)e^{i2\theta }]$, which contributes to the plasmonic field of the center point by ${\vec{F}}_{1}={\vec{e}}_{z}\sqrt{2}\pi E_{0}r_{0}e^{-k_{z}z}e^{ik_{r}r_{0}}J_{0}\left(0\right)$. The plasmonic field resulting from the “1” input state of an input signal is $i{\vec{e}}_{z}\sqrt{2}\pi E_{0}r_{0}e^{-k_{z}z}e^{ik_{r}r_{0}}[J_{0}\left(-k_{r}R\right)+J_{2}\left(-k_{r}R\right)e^{i2\theta }]$, which contributes to the plasmonic field of the center point by $i{\vec{F}}_{1}=i{\vec{e}}_{z}\sqrt{2}\pi E_{0}r_{0}e^{-k_{z}z}e^{ik_{r}r_{0}}J_{0}\left(0\right)$. Thus, the plasmonic field at the center point under input states “00”, “10/01”, and “11” are respective $2{\vec{F}}_{1}$, $(1+i){\vec{F}}_{1}$ and $2i{\vec{F}}_{1}$, corresponding to normalized intensity 1, 0.5 and 1, respectively.

## 4. Conclusions

In conclusion, we have put forward a vortex beams encoded scheme to achieve all-optical logic gates based on nano-ring plasmonic antennas. OR, AND, NOT, NOR, NAND and XNOR logic gates are designed and discussed. Since our scheme is compatible with vortex beams, it may have potential applications in vortex beam-based all-optical computing. In addition, since the size of the nano-ring plasmonic antenna can be designed on the scale of several wavelengths of the plasma wave, our scheme is very suitable for device miniaturization and integration.

## Figures and Tables

**Table 1:** OR and AND logic gates.

Input States of Signal 1	Input States of Signal 2	Normalized Intensity of the Center Point
1	1	1
1	0	0.25
0	1	0.25
0	0	0

**Table 2:** NOT logic gate.

Input States	Normalized Intensity of the Center Point
0	1
1	0

**Table 3:** NOR and NAND logic gates.

Input States of Signal 1	Input States of Signal 2	Normalized Intensity of the Center Point
0	0	1
1	0	0.25
0	1	0.25
1	1	0

**Table 4:** XNOR logic gate.

Input States of Signal 1	Input States of Signal 2	Normalized Intensity of the Center Point
0	0	1
1	0	0.5
0	1	0.5
1	1	1
